# The Scope of Usage-Based Theory

**DOI:** 10.3389/fpsyg.2013.00255

**Published:** 2013-05-08

**Authors:** Paul Ibbotson

**Affiliations:** ^1^The Open UniversityUK

**Keywords:** usage-based, language processing, language-acquisition, typology, cultural learning

## Abstract

Usage-based approaches typically draw on a relatively small set of cognitive processes, such as categorization, analogy, and chunking to explain language structure and function. The goal of this paper is to first review the extent to which the “cognitive commitment” of usage-based theory has had success in explaining empirical findings across domains, including language acquisition, processing, and typology. We then look at the overall strengths and weaknesses of usage-based theory and highlight where there are significant debates. Finally, we draw special attention to a set of culturally generated structural patterns that seem to lie beyond the explanation of core usage-based cognitive processes. In this context we draw a distinction between cognition *permitting* language structure vs. cognition *entailing* language structure. As well as addressing the need for greater clarity on the mechanisms of generalizations and the fundamental units of grammar, we suggest that integrating culturally generated structures within existing cognitive models of use will generate tighter predictions about how language works.

“Don’t ask for the meaning; ask for the use” – *Wittgenstein, Philosophical Investigations*

Linguistic theory has a lot to explain. First, the structure of language emerges over vastly different time scales: the split-second processes of producing and comprehending speech; the years an individual takes to construct their language; the centuries over which languages evolve. Second, there is the sheer diversity of forms languages take. Despite the rate at which languages are disappearing, a person would have to walk less than 50 miles on average to meet a new language if all they were equally distributed over the inhabitable world. Compare this with our closest living evolutionary cousins. The odd cultural variation in handshake in Chimpanzees is impressive but it does not really compete with over 6000 ways of saying “hello” (Foley and Lahr, 2011). Third, language is a complex adaptive system (Bybee, 2010). This means the language people know changes and reorganizes itself in response to multiple competing factors. The nested feedback loops this sets up defy any simple input-output analysis, ratcheting up the complexity further.

The structure of this paper is as follows. First, we give a brief outline of the core cognitive processes that underpin the usage-based approach. Second, we examine the extent to a usage-based cognitive framework predicts empirical findings in language acquisition, processing, and typology – domains that are not usually not brought together in this way. One criterion to judge the strength of usage-based theory is to examine whether it has made subtle predictions across a range of domains. Converging evidence from separate domains that employ a vast range of methodologies and occupy a wide range of positions on the theoretical spectrum would provide particularly strong support. Third, we briefly discuss the philosophical implications of adopting a usage-based approach before reflecting on what usage-based theory does well, what it does less well, and where there are significant points of disagreement pointing to new lines of research. Finally, we present findings from the linguistic anthropological literature, looking at ways in which culture may constrain linguistic structure. We suggest a challenging new line of research will be to ground culturally dependent structures within existing cognitive models of use.

## The Usage-Based Approach

Despite the daunting scope of linguistic phenomena begging an explanation, usage-based theories of language representation have a simple overarching approach. Whether the focus is on language processing, acquisition, or change, knowledge of a language is based in knowledge of actual usage and generalizations made over usage events (Langacker, 1987, 1991; Croft, 1991; Givón, 1995; Tomasello, 2003; Goldberg, 2006; Bybee, 2010). For usage-based theories the complexity of language emerges not as a result of a language-specific instinct but through the interaction of cognition and use.

These broad assumptions are in contrast with those of the generativist and structural traditions who analyze language as a static or synchronic system, self-contained, and autonomous from the cognitive and social matrix of language use. The aim here is not to detail the case *against* generativist universal grammar (plenty of that criticism exists elsewhere, e.g., Tomasello, 2004; Christiansen and Chater, 2008; Evans and Levinson, 2009; see Ambridge and Lieven, 2011 for contrasting theoretical approaches to language acquisition). Here we devote more space to the strengths and weaknesses of usage-based theory and point to where there might be new theoretical ground to break. One thing is worth briefly stating here though. Much theoretical work in generativist linguistics followed from the assumption that it was impossible for a child to acquire the rules of their grammar *with the tools of Behaviorism*. Few developmental psychologists would disagree with this but equally, after 50 years of research into the social-cognitive abilities of children, few developmental psychologists would define the resources available to the child in merely Behaviorist terms. The section on language acquisition and cultural use outlines the nature of what some of these resources are – although it is a point of debate whether one is convinced that these resources provide a complete account of language acquisition.

The usage-based position is closely allied with that of Cognitive Linguistics and Construction Grammar where the fundamental linguistic unit is the construction: a meaningful symbolic assembly used for the purpose of signally relevant communicative intentions. Syntactic schemas, idioms, morphology, word classes, and lexical items are all treated as constructions that vary along a continuum of specificity (Langacker, 1987; Fillmore et al., 1988). If one accepts the premise that language is like this, the developmental implications are quite profound: if children can learn idiosyncratic yet productive constructions (e.g., *Her go to the ballet!* *My mother-in-law ride a bike!*), then why can they not learn the more canonical ones in the same way? In the cognitive linguistic/construction grammar approach, a grammar is more than a list of constructions however. The organization and productivity of language is understood as the result of analogies between the form and/or meaning in a structured inventory of constructions. For example, the phrases *the taller they come the harder they fall*, *the more the merrier*, *the older they get the cuter they ain’t* are all variations on an underlying theme or productive schema; *the* X-*er* *the* Y-*er*. Moreover, there are no core/periphery, competence/performance distinctions of generativist theory – it is constructions and use all the way down (and up). This very brief characterization of usage-based approaches gives a flavor of what usage-based theorists think language *is*. We now turn to the cognitive processes that are thought to generate linguistic structure.

## Usage-Based Processes

Bybee (2010), a key architect of usage-based theory, identifies several cognitive processes that influence the use and development of linguistic structure (i) categorization; identifying tokens as an instances of a particular type (ii) chunking; the formation of sequential units through repetition or practice (iii) rich memory; the storage of detailed information from experience (iv) analogy; mapping of an existing structural pattern onto a novel instance, and (v) cross-modal association; the cognitive capacity to link form and meaning. Thus there is a strong “cognitive commitment” to explaining linguistic structure. As Taylor puts it, “the general thrust of the cognitive linguistics enterprise is to render accounts of syntax, morphology, phonology, and word meaning consonant with aspects of cognition which are well documented, or at least highly plausible, and which may manifest in non-linguistic (Taylor, 2002, p. 9).

As an example of this commitment, Ibbotson et al. (2012) looked at whether we can “import” what we know about categorization of non-linguistic stimuli to explain language use. In a classic study by Franks and Bransford (1971) they argued that a prototype comprises of a maximal number of features common to the category, often “averaged” across exemplars. They constructed stimuli by combining geometric forms such as circles, stars, and triangles into structured groups of various kinds. Some of these were then shown to participants who were then later asked whether they recognized these and other shapes they had not seen previously. Importantly, one of the exemplars shown at test contained all of the geometric forms together, an exemplar that had actually never been shown previously (but could be considered the prototype if all of the experienced exemplars were averaged). The participants not only thought that they had seen this prototype, but they were actually more confident that they had seen it than the other previously seen exemplars (or distracter items which they had not seen). Note that these effects were established for an *ad hoc* non-linguistic category. Ibbotson et al. found the same pattern of findings – misrecognition of prototypes – extends to the transitive argument-structure construction, a fundamental building block present in one form or another in all of the world’s languages (Hopper and Thompson, 1980; Næss, 2007). They argued that this shows abstract linguistic categories behave in similar ways to non-linguistic categories, for example, by showing graded membership of a category (see also Ibbotson and Tomasello, 2009 for a cross-linguistic, developmental perspective).

**Figure 1:**
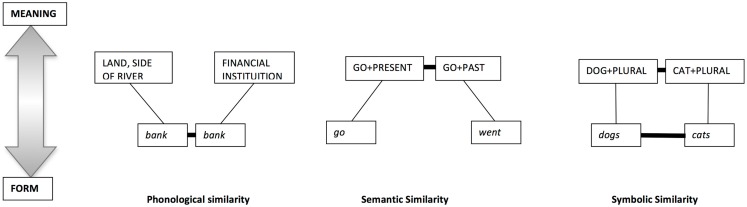
**Constructions grouped together on the basis of similarity of form and/or function (adapted from Croft and Cruse, 2004)**.

Focusing on analogy for the moment, in usage-based theory, constructions can be analogous in form and/or meaning (Figure 1), and there is good evidence that the alignment of relational structure and mapping between representations is a fundamental psychological process that underpins forming these abstract connections (Goldstone et al., 1991; Goldstone, 1994; Goldstone and Medin, 1994; Gentner and Markman, 1995).

There is quite a lot of evidence that people, including young children, focus on these kinds of relations in making analogies across linguistic constructions, with some of the most important being the meaning of the words involved, especially the verbs, and the spatial, temporal, and causal relations they encode (e.g., Gentner and Markman, 1997; Gentner and Medina, 1998; Tomasello, 2003). Bod (2009) showed how a computational learning algorithm was able use structural analogy, probabilistically, to mimic children’s language development from item-based constructions to abstract constructions, including simulation of some of the errors made by children in producing complex questions. In usage-based theory, analogy also operates in a more abstract sense, by extending the prototypical meaning of constructions. For example, the meaning of the ditransitive construction is closely associated with “transfer of possession” as in “*John gave Mary a goat*.” Metaphorical extensions of this pattern, such as “*John gave the goat a kiss*” or even “*Cry me a river*” are understood by analogy to the core meaning of the construction from which they were extended, which in the case of the ditransitive is something like “X causes Y to receive Z” (Goldberg, 2006).

The relative frequency of items in a corpus obviously plays a key role in many usage-based processes. Items that consistently co-occur together in the speech stream and are consistently used for the same function face a pressure to become *automatized*, in a manner that is similar to those which occur in a variety of non-linguistic sensory-motor skills. Thus, *going to* faces pressure to be compressed or chunked to *gonna*. The ultimate message compression strategy would be to say nothing at all, thus, at many different levels – grammatical, lexical, phonological – the linguistic system is trying to balance the trade-off between the amount of linguistic signal provided for a given message and the expectedness of that message (Jaeger and Tily, 2011). Irregular patterns can survive in the language because they are frequent enough to be learned and used on their own, whereas items and constructions that are less frequent tend to get regularized by pattern-seeking children or, in the extreme case, they drop out of use as children do not get enough exposure to them. The more a linguistic unit is established as a cognitive routine or “rehearsed” in the mind of the speaker, the more it is said to be *entrenched* (Langacker, 1987). Entrenchment is a matter of degree and essentially amounts to strengthening whatever response the system makes to the inputs that it receives (Hebb, 1949; Allport, 1985). The converse of this is that extended periods of disuse will weaken representations. For example, young infants are able to discriminate a wider range of sounds that are present in their native language (Aslin et al., 1981). Once this entrenchment is established as a routine it can be difficult to reverse. For example, Japanese speakers find it difficult to discriminate between /r/ and /l/ because it activates a single representation, whereas for English speakers the two representations remain separately entrenched (Munakata and McClelland, 2003).

We will now look at the extent to which core usage-based cognitive processes – like categorization, analogy, and chunking – can explain the empirical findings in language acquisition, processing, and change. Obviously these are all huge areas of research in their own right and there is no way to test usage-based theory against every finding. In the space we have, we try to focus on studies that make key points and are indicative of the wider literature.

## Language Acquisition and Use

The good news for children is that they usually do not have to invent ways of communicating *de novo*. The language they are born into is very likely to be the result of a long history of cumulative cultural evolution and so provides them with off-the-peg solutions to communicative problems. The bad news is that they have to learn what these are.

### Usage patterns

**Figure 2:**
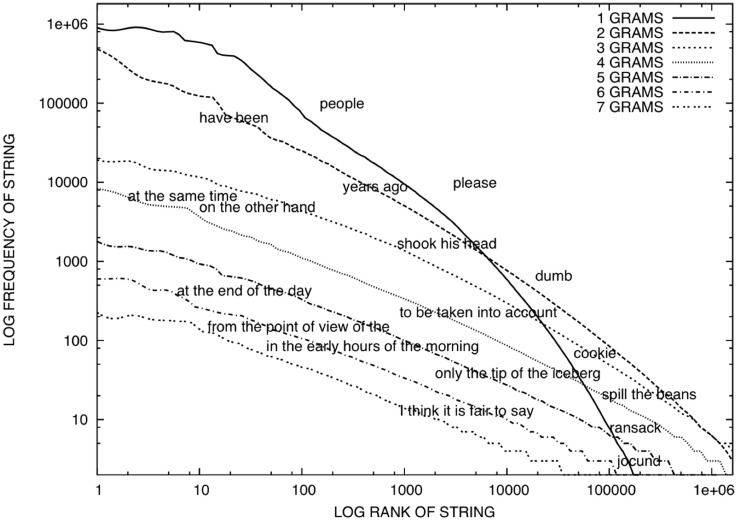
**From Bannard and Lieven (2009)**. The figure plots the natural logarithm of the frequency of each string of words encountered against the natural logarithm of the position of each string in a ranked list of these substrings. Words are taken from the 89 million word written component of the British National Corpus (Burnard, 2000).

For a five-word string (e.g., *I like your green cheese*), with 20 words that can fill each slot, there are 3,200,000 possible five-word sentences (20^5^). An important point for both processing and acquisition is that, in natural language the probability of encountering any given word or any combination of these words is not equal. Specifically, there are a few forms that are encountered relatively frequently whereas most are encountered rarely (Zipf, 1935/1965). As Shannon pointed out, this kind of redundancy allows recovery of meaning xvxn whxn thx sxgnxl xs nxxsy (Shannon, 1951). Interestingly, this is true not only for letter sequences and words (1 GRAM) but for sequences of words too (GRAM > 1), Figure 2.

This formulaticity in language has consequences for learning. Item-based or exemplar-based approaches to learning recognize that a large part of a person’s language knowledge is constructed from rather specific pieces of language, grounded in the expressions people actually use during their everyday interactions. These items or exemplars are stored in a way that is not far removed (in terms of schematicity) from the way in which people encounter them in natural discourse. Focusing on Child Directed Speech, Cameron-Faulkner et al. (2003) found that a small number of semi-formulaic frames account for a large amount of the data. Frames like *Where’s the* X? *I wanna* X, *More* X, *It’s a* X, *I’m* X-*ing it*, *Put* X *here*, *Mommy’s* X-*ing it*, *Let’s* X *it*, *Throw* X, X *gone*, *I* X-*ed it*, *Sit on the* X, *Open* X, X *here*, *There’s a* X, X *broken*. Decades of research support the general idea that children used these islands of reliability when they are schematizing patterns in their language (e.g., Tomasello, 2003; Ambridge and Lieven, 2011).

Pronouns are a particularly important source of reliability in the input as children hear lots of these examples. Ibbotson et al. (2011) systematically varied constructional cues (NP *is verbing* NP vs. NP *is getting verbed by* NP) and case-marking cues on English pronouns (*he/she* vs. *her/him* vs. *it*). These can be configured as different “pronoun-frames,” for example (NP_nominative_ – Verb – NP_accusative_) vs. (NP_neuter_ – Verb – NP_accusative_). In a pointing comprehension test, both 2- and 3-year-olds used the case-marking on the pronoun frames to help them comprehend the sentences; this despite the fact that English has a relatively restricted case system compared to other languages like Finnish and Turkish. It shows even very young English children are sensitive to complex patterns of use – in this case comprehending the “value added” by case in marking the semantic roles of transitives over and above that of word order and other constructional cues. As Goldin-Meadow (2007) has pointed out pronoun frames may also have special status as they essentially act as deictic/pointing expressions. A child can than map the gestural productivity of a point – which can refer to anything in the world – onto the referential flexibility of lexical pronouns.

This item-based view does not necessarily entail that children do not, at some point, understand phrases like *Susan tickles Mary* or words like *hands* as exemplars of a more general pattern or schema, say X verbs Y or plural = noun + s. It is a claim about how children might get to this point of development and how information is stored in the mind of the speaker. Exemplar-based approaches are therefore as much about how language is represented as about the process of learning itself. In the usage-based view of language acquisition, children first acquire a number of lexical schemas or semi-formalic frames, e.g., X *hits* Y, X *kisses* Y, X *pushes* Y, X *pulls* Y, then by forming analogies between the roles that participants are playing in these events, these constructions eventually coalesce into a general abstract construction, e.g., X Verb_transitive_ Y (Tomasello, 2003; Bannard et al., 2009; Ibbotson, 2011).

Several researchers have attempted to simulate the process of moving from items to schemas in more mechanistic terms with computational models. For example, Bannard et al. (2009) built a model of acquisition that begins with children learning concrete words (exemplars) and phrases, with no categories or grammatical abstractions of any kind. That model was then compared to a model using grammatical categories and they found that the category-less model provided the best account of 2-year-old children’s early language. When categorical knowledge, comprising nouns and verbs, was entered into this item-based model, performance actually got worse. Importantly and in contrast, at 3 years of age adding grammatical categories such as nouns and verbs helped the performance, suggesting that these categories emerged around the third year of life. Another important finding was that at 2 years of age children’s item-based grammars were very different from one another, whereas by 3 years of age children’s more category-based grammars were more similar to one another, as the children all began converging on, in this case, English grammar (see also Bod, 2009 for a structural analogy model). Mechanisms of analogy and generalizations are a key area of research in usage-based theory and are discussed later in the sections on strengths, weaknesses, and challenges.

Having introduced moving from items to schemas, usage-based theory needs some proposal of how to constrain generalizations so that children arrive at the same conventions as adult do. The three main suggestions that would help children rule out some overgeneralizations are, in one way or another, all based on patterns of use (see Ambridge et al., 2013 for more detailed supporting evidence for each).

1. Entrenchment (Braine and Brooks, 1995): the more often a child hears a verb in a particular syntactic context (e.g., *I suggested the idea to him*) the less likely they are to use it in a new context they haven’t heard it in (e.g., *I suggested him the idea; Ambridge et al., 2012a; see also Perfors et al., 2010 who use hierarchical Bayesian framework to model this kind of generalization).
2. Pre-emption (Goldberg, 2006): if a child repeatedly hears a verb in a construction (e.g., *I filled the cup with water*) that serves the same communicative function as a possible unattested generalization (e.g., *I filled water into the cup), then the child infers that the generalization is not available (Ambridge et al., 2012b).
3. Construction semantics (Pinker, 1989): constructions are associated with particular meanings (e.g., the transitive causative with direct external causation). As children refine this knowledge, they will cease to insert verbs that do not bear these meaning elements into the construction (e.g., *The joke giggled him; Ambridge et al., 2011).

### What words refer to and usage patterns

**Figure 3:**
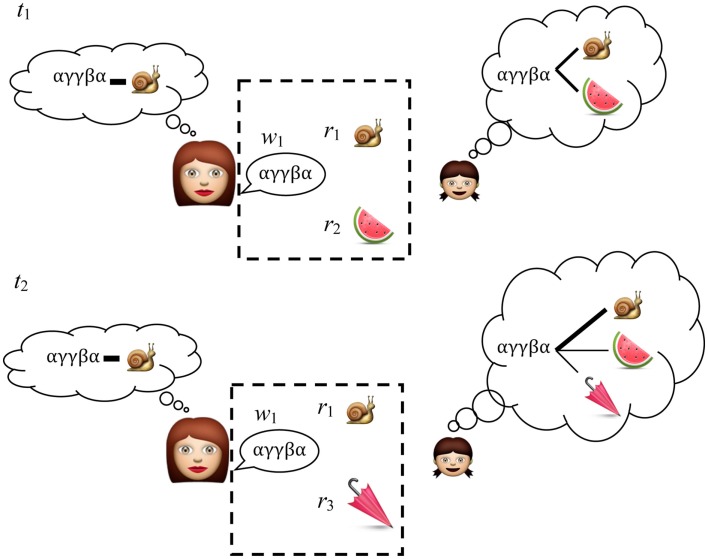
**An adult chooses a linguistic expression w_1_ associated with concept r_1_ that they want to communicate**. At t_1_ the child doesn’t know whether the novel word w_1_ refers to r_1_ or r_2_ and for now the best she can do is remember the associations between the scene and the words. At t_2_ she hears w_1_ with one object r_1_ familiar from t_1_ and one new object r_3_. Cross-situational learning works by repeatedly recording the associations between language and the context in which it is used. Over time, the signal (an intentional word-referent pair) is more strongly represented than the noise (an unintentional word-referent pair). Items that appear in the hashed box are the raw data on which the child makes the cross-situational associations.

Interestingly the issue of determining a word’s referent has often been presented as one shot learning problem (following Quine, 1960) – evidently not the same problem children actually face. By using invariant properties in the word-to-world mapping across multiple situations, it has been shown children reduce the degrees of freedom as to what nouns and verbs might mean, Figure 3 (Siskind, 1996; Smith and Yu, 2008; Scott and Fisher, 2012).

However powerful cross-situational learning is, it undoubtedly operates in combination with other cues to meaning. Yu and Ballard (2007) found that a computational model of word-learning performed better when it used social information (joint attention and prosody) in combination with statistical cues (cross-situational learning) than when it relied on purely statistical information alone (see also Frank et al., 2009 for the integration of pragmatic and statistical cues). Once the child has a foothold in the language, the context of the novel word gives a clue as to what *kind* of word it might be (Gleitman, 1990; Pinker, 1994). For example, the usage patterns in English hint at the following possibilities for *pum* (MacWhinney, 2005):

(1) *Here is a pum* (count noun).
(2) *Here is Pum* (proper noun).
(3) *I am pumming* (intransitive verb).
(4) *I pummed the duck* [transitive (causative) verb].
(5) *I need some pum* (mass noun).
(6) *This is the pum one* (adjective).

Eight-month-old infants are sensitive to the statistical tendency that transitional probabilities are generally higher within words than across words, helping them to find “words in a sea of sounds” (Saffran et al., 1996; Saffran, 2001). Children are also able to discover syntactic regularities between categories of words as well as the statistical regularities in sound patterns (Marcus et al., 1999). By 12 months, infants can use their newly discovered word boundaries to discover regularities in the syntactic distributions of a novel word-string grammar (Saffran and Wilson, 2003). And by 15 months old, infants are able to combine multiple cues in order to learn morphophonological paradigmatic constraints (Gerken et al., 2005). Note the domain-generality of this statistical learning ability. 2-month-olds infants are sensitive to transitional probabilities between visual shape sequences (Kirkham et al., 2002) thus infants have already build up considerable expertize in statistical learning by the time these linguistic effects appear.

### Adult’s use and children’s use

As usage-based theories would predict, errors in child speech, the age at which structures are acquired, and the sequence that language emerges can all be predicted on the basis of usage patterns in the input. For example,

- The acquisition order of *wh*-questions (*What, Where, When, Why, How*) is predicted from the frequency with which particular *wh*-words and verbs occur in children’s input (Rowland and Pine, 2000; Rowland et al., 2003).
- Children’s proportional use of *me-for-I* errors (e.g., when the child says “me do it”) correlates with their caregivers’ proportional use of *me* in certain contexts (e.g., “let me do it” (Kirjavainen et al., 2009).
- The pattern of negator emergence (*no* → *not* → ’*nt*) follows the frequency of negators in the input; that is negators used frequently in the input are the first to emerge in the child’s speech (Cameron-Faulkner et al., 2007).
- Children’s willingness to construct a productive schema reflects their experience with analogous lexical patterns in the input (Bannard and Matthews, 2010).
- Children are less likely to make errors on sentences containing high-frequency verbs compared low frequency verbs (Brooks and Tomasello, 1999; Theakston, 2004; Ambridge et al., 2008).

Overall it seems there is good evidence to support the usage-based prediction that language structure emerges in ontogeny out of experience (viz. use) and when a child uses core usage-based cognitive processes – categorization, analogy, form-meaning mapping, chunking, exemplar/item-based representations – to find and use communicatively meaningful units.

## Language Processing and Use

In this section we look at how the “cognitive commitment” of usage-based theory has been used to explain findings in language processing.

### How words are stored and used

The approach from the generativist tradition has been to propose a fundamental division of labor between linguistic units and the rules that combine them into acceptable combinations, for example, singular *muggle* + plural marker *s* = plural *muggles* (e.g., Pinker, 1999). The usage-based view is that productivity is a result of knowledge generalized over usage events. Pluralizing a new word like *muggle* is by analogy to a relevant class of linguistic experience. Even regular forms, such as *books*, *dogs*, and *cars*, if encountered frequently enough are represented and stored as chunks. This process combines several of the core cognitive principles we are interested in here – categorization, analogy, chunking, and a rich memory for exemplars so plural formation is a good to test case for usage-based theory.

The well-established and unsurprising fact is that people are quicker to recognize high-frequency words; the more often a word is encountered the more entrenched that representation is and the more easily it is retrieved. The interesting question here is whether peoples’ response times are influenced by the frequency of the plural word form, e.g., *books* or by the combined frequency of the forms *book* + *books*. If plurals are stored separately, as predicted by usage-based approaches, we should expect that the frequency of the plural to be the crucial factor. Sereno and Jongman (1997) flashed words interspersed with non-words onto a monitor and asked participants whether the word was a word or not. They created two lists of nouns which were matched for their overall frequency in the language, but differed with respect to the ratio of singular to plural forms. List A contained words which predominantly occurred in the singular (*island*, *river*, *kitchen*, *village*) whereas list B contained forms that occurred equally often in singular and plural forms (*statement*, *window*, *expense*, *error*). In support of the usage-based hypothesis, singulars from List A were recognized more quickly than singulars from list B and plurals from group B were recognized more quickly than plurals from list A. Further research confirms their conclusions for plural formation (e.g., Baayen et al., 1997; Alegre and Gordon, 1999) and the same argument, namely, that high-frequency forms tend to be stored and processed as wholes, has also been extended to cover past tense formation in English (Bybee, 1999). The overall picture is one of a rich memory for exemplars, whereby sequences of units that frequently co-occur cohere to form more complex chunks (Reali and Christiansen, 2007; Ellis et al., 2008; Kapatsinski and Radicke, 2009).

This sensitivity to detailed distributional information exists not just at the levels of words but on many levels of linguistic analysis; from words to larger chunks to phrases (see Diessel, 2007 and Ellis, 2002 for reviews). For example, in a series of studies, Arnon and Neal (2010) showed that comprehenders are sensitive to the frequencies of compositional four-word phrases (e.g., *don’t have to worry*) such that more frequent phrases were processed faster (cf. Bannard and Matthews, 2010 for an interesting comparison with children). The effect was not reducible to the frequency of the individual words or substrings and was observed across the entire frequency range (for low-, mid-, and high-frequency phrases). They conclude that comprehenders seem to learn and store frequency information about multi-word phrases. These findings adds to the growing processing literature that broadly supports the usage-based position: processing models capture and predict multi-level frequency effects and support accounts where linguistic knowledge consists of patterns of varying sizes and levels of abstraction.

Overall the psycholinguistic evidence supports the idea that representations are instantiated in a vast network of structured knowledge, which is encyclopedia-like in nature and in scope (see review by Elman, 2009). Knowledge of fairly specific (and sometimes idiosyncratic) aspects of verbal usage patterns is available and recruited early in sentence processing. For example:

- Sentences of the type *the boy heard the story was interesting* have structural ambiguity. At the point of parsing *the story*, it could be the direct object (DO) of *heard* or it could be the subject noun of a sentential complement (SC), as it was in the above example. Hare et al. (2003, 2004) showed that it was possible to induce a DO or SC readings of the same verb on the basis of whether the preverbal context was associated with one reading or another (as determined from a corpus of natural speech). The message here is that people are sensitive to statistical patterns of usage that are associated with specific usages of an individual verb.
- McRae et al. (1998) showed that *The cop arrested* promoted a main verb reading (*the cop arrested X*) over a reduced relative *(the cop arrested by the X*). Conversely, *the criminal arrested* promoted a reduced relative over a main verb reading. McRae concludes that the thematic role specifications for verbs must go beyond the traditional categories like agent, patient instrument, beneficiary to include very detailed information about the preferred fillers of the role and the prototypical events in which they take part (in the above example that criminals are more likely to be arrested than do the arresting and vice versa for cops).
- Homonyms prime each other, for example bank (financial institution) momentarily activates the representation bank (land side of river), showing that linguistic knowledge is organized along phonological as well as the semantic dimensions (see Figure 1) (Swinney, 1979; Seidenberg et al., 1982).

All of this argues against a serial account of sentence processing where syntax proposes a structural interpretation for semantics to subsequently cash out (Frazier, 1987, 1989; Clifton and Frazier, 1989). Moreover, verbal knowledge is sensitive to contingencies (usage patterns) that hold across agents, aspect, instruments, event knowledge, and broader contextual discourse, so that altering any one may skew the construal of what is communicated. It seems unlikely then that syntactic knowledge is encapsulated from semantic, pragmatic, and world knowledge (e.g., Fodor, 1995). Rather, the evidence is converging on the idea of words as probabilistic cues that point to a conceptual space of possibilities, which is revised and honed as the discourse unfolds between speakers.

## Language Diversity and Use

Like all scientific enterprises, linguistics is in the business of finding and explaining patterns. Why are languages the way they are, rather than some other way they could be? Again, we will examine the extent to which the cognitive commitment of usage-based linguistics has been able to account for findings at this level of description. Word order provides a good example. The world’s languages can be categorized on the basis of how they order the basic constituents of a sentence – subject, object, and verb – which function to coordinate “who did what to whom.” Of the six logically possible word orders this creates, some are much more prevalent than others. A survey of 402 languages reveals that the majority of languages favor either SOV (44.78%) or SVO (41.79%) with the other possibilities – VSO (9.20%), VOS (2.99%), OVS (1.24%), or OSV (0.00%) – significantly less popular (Tomlin, 1986).

There are several mechanisms that could be at work here to create this typological distribution. For example, we know there is a general preference to speak of given information before information that is new to the discourse (MacWhinney and Bates, 1978; Bock and Irwin, 1980; Prat-Sala and Branigan, 2000; Ferreira and Yoshita, 2003), a preference for shorter dependency length (Hawkins, 1994, 2007) and a preference for efficient information transfer (Maurits et al., 2010). Over historical time frames the serial position becomes grammaticalized into subject, verb, and object position, hence Givón’s (1979, p. 208–209) aphorism “today’s syntax is yesterday’s pragmatic discourse.” However, this doesn’t explain why *any particular* language looks the way it does; here we need a historical perspective. Dunn et al. (2011) used computational phylogenetics to show that typological variation in word order could be explained as a function of the iterated learning processes of cultural evolution. Croft et al. (2011) provide criticism of their methodology – the absence of any Type II error analysis to assess the rate of false negatives, the absence of contact effects and the nature of the phylogenies used – although they are in favor of the general approach. These criticisms do not undermine the wider point, namely, that popularity of word orders across languages can be explained in terms of general cognitive principles (e.g., pressure for iconicity of form and function, or concise representation of salient/frequent concepts; see section below). *Why any particular language* has come to the combination of solutions it has, needs reference to its historical antecedents/evolution (Bybee, 2010). To preface a distinction we return to in the final section, this can be thought of as the difference between cognition *permitting* a particular language vs. cognition *entailing* a particular language.

In the context of historical antecedents, an analogy with biological evolution may be informative. Evolution has to work with what it’s got, cumulatively tinkering with solutions to problems that have worked well enough for previous generations, but which might not be considered “ideal” if one could start afresh (e.g., the backward installed retina in humans). An evolutionary biologist could ask the question “Why do we only see the mammals we do given all the possible mammals that could exist but don’t?” For the biologist, different mammals could be thought of as a record of competing motivations, that have over time explored some of the space of what is physiological plausible for a mammal to be. For a particular feature of the animal’s development, for example the skeleton, a bat could be thought of as occupying one corner of this space while an elephant skeleton is in another – extreme variations on an underlying theme. Different languages are also a history of competing motivations that have explored some of the space of what is communicatively possible. Over time languages have radiated to different points in this space. For a particular feature of the language development, for example the sound system, in one corner there might be a three-vowel system (e.g., Greenlandic, an Eskimo-Aleut language) while in another corner is a language with 24 vowels (e.g., !Xu, a Khoisan language). Importantly, as language is a complex adaptive system, evolving toward an extreme in one direction will have functional consequences for the system as a whole. Just as a bat skeleton will place certain functional demands on the rest of its physiology, so it is with language. In languages with freer word order, the communicative work that is done by a fixed word order in other languages must be picked up by other aspects of the system, e.g., morphology and pragmatic inference. The analogy with biological evolution might be useful in another way. The eye has independently evolved in several different species, converging on a similar solution to a similar engineering problem. The major nuts and bolts of grammar (e.g., word-order, case-agreement, tense-aspect) that appear time after time in the world’s languages could be thought of as historically popular solutions to similar communicative and coordination problems, such as sharing, requesting, and informing. Having set out the general usage-based picture with respect to the role of history, we take a closer look at the cognitive constraints on cross-linguistic structure and assess whether this helps explain typological similarity and diversity.

### Explaining similarity and diversity

The idea that languages are mostly alike has been undermined by the growing amount of evidence that languages are actually quite distinct (e.g., Bowerman and Choi, 2001; Evans and Levinson, 2009). The remaining similarities can typically be explained with respect to four domain-general classes of constraint which simultaneously act to shape language: perceptuo-motor factors, cognitive limitations on learning and processing, constraints from thought, and pragmatic constraints (e.g., Goldberg, 2006; Diessel, 2007, 2011; Ambridge and Goldberg, 2008; Christiansen and Chater, 2008).

Information-theoretic approaches to communicative efficiency have shown how human cognitive abilities directly or indirectly constrain the space of possible grammars (for a recent review of this, see Jaeger and Tily, 2011). Processing difficulty in comprehension and production can arise when the sentence overloads the memory systems or contains combinations of words which are infrequent, unexpected or for which there are competing cues. There are several ways in which on-line processing difficulty and a drive for communicative efficiency might constrain the typological distribution of grammars, for example, in acquisition, less complex forms might be learned preferentially due to higher frequency in the input. The processing approach to typology suggests that grammars preferentially conventionalize linguistic structures that are processed more efficiently (Hawkins, 1994, 2007). Evidence from English and German suggests that the average dependency length (known to correlate with processing difficulty; e.g., Gibson, 1998) in these languages is close to the theoretical minimum and far below what would be expected by chance (e.g., Gildea and Temperley, 2010). Moreover, work investigating learner’s preferences in the artificial language learning paradigm show that (a) learners of experimentally designed languages acquire typologically frequent patterns more easily than typologically infrequent patterns (Christiansen, 2000; Finley and Badecker, 2008; Hupp et al., 2009, St Clair et al., 2009); and, (b) learners restructure the input they receive shifting the acquired language toward typologically natural patterns (Fedzechkina et al., 2011; Culbertson et al., 2012).

The sheer diversity of forms that languages take presents all theories with a massive challenge. For example, as far as know, Pirahã is the only language in the world to use the sound of a voiced linguolabial lateral double-flap (Everett, 2012). As Everett points out, all theories of language would struggle to predict both its existence *and* rarity. In fact, it is much easier to identify common patterns of language change than it is to find cross-linguistic similarities (Bybee, 2010). Usage-based theories argue this is because there is a universal set of cognitive processes underlying cross-linguistic cycles (e.g., grammaticization, pidginization, and creolization) and the fact that all languages have to pass through the bottleneck of what is learnable (Bybee et al., 1994; Diessel, 2007, 2011; Heine and Kuteva, 2007; Christiansen and Chater, 2008).

### Speaking the same language

It is uncontroversial that, as a function of linguistic experience, different speakers of the same language have different dialects, vocabularies, phrases, idioms, accents, and so on. For some reason, many linguists from all theoretical persuasions have resisted applying this logic to grammar. There is a wide spread assumption that adults are converging on the same grammar when they learn a particular language (Seidenberg, 1997, p. 9; Crain and Lillo-Martin, 1999, p. 1600; Nowak et al., 2001, p. 114). Dąbrowska (2012) challenges the prevailing wisdom by showing that different speakers have different grammars as a function of their individual usage histories. For example, the inflections on the polish dative are highly predictable and so in theory all adults should share the same intuitions: -*owi* for masculine nouns, -*u* for neuter nouns, and -*e*, -*i*/*y* for feminines. Dąbrowska (2008) asked adults to use nonce words, presented in the nominative, in contexts that required the dative. Individual scores on the inflection task ranged from 29 to 100% correct. The usage-based hypothesis would predict familiarity with noun exemplars (and thus familiarity with inflecting them) would correlate with the productivity of a particular morphological schema. This is what was found, with performance on the task positively correlated with vocabulary (*r* = 0.65, *p* < 0.001), which is itself correlated strongly in this experiment with years spent in formal education (*r* = 0.72, *p* < 0.001).

Analogous adult variation in comprehension has been demonstrated with the active-passive distinction (Dąbrowska and Street, 2006) and quantifiers in English (Brooks and Sekerina, 2006). The fact that both passive and quantifier performance could be improved with training (Street and Da?browska, 2010) underlies the role of use. Dąbrowska (2012) concludes that the results from English and Polish show that some speakers extract only fairly specific, local, and lexically based generalization whereas others are able to be more flexible and apply more abstract patterns. In general, this ability was tightly correlated with use, in a particular a more varied linguistic experience, which in this case was often education-related. It is worth noting that in the terminology of generativist grammar, we are talking about core competence here: passives, complex sentences, quantifiers, and morphological inflection. Different levels of grammatical attainment in healthy adults – even in “core competency” – are what one would expect if people build their grammar bottom-up as a function of their linguistic experience.

## Philosophical Foundations and Use

Is there any broad philosophy that a usage-based approach to language follows? One candidate would be a non-essentialist view of describing the world (Janicki, 2006; Goldberg, 2009). Plato used the Greek word logos to mean the “ultimate essence”; the underlying reality or true nature of a thing that one cannot observe directly. So for instance, we understand circles as circles with respect to an abstract, unchanging ideal of what a circle is, laid out by well-defined criteria, such as “its area is equal to π*r*^2^.” With this mindset, one can then sensibly ask what is the essential quiddity or “whatness” for all sorts of things; “what is beauty,” “what is truth,” and so on. This kind of essentialist thinking was reincarnated in the philosophy of the Logical Positivists who sought to verify whether propositions were true or false with respect to the underlying logical structure of language. If we could only do this, as Leibniz had hoped, then “two philosophers who disagree about a point should, instead of arguing fruitlessly and endlessly, be able to take out their pencils, sit down amicably at their desks, and say ‘Let us calculate.”’

In one of the most famous (and honest) about-turns in intellectual history, Wittgenstein (1953/2001) completely abandoned his involvement in this approach, no longer so interested in the relationship between meaning and truth as that between meaning and use – hence the quote at the beginning of this article. The change in his thinking can be summarized in the change of his metaphors; from language as a picture (true propositions are those that picture the world as it is) to language as a tool. Tools are defined by what they do, how they are used. He realized that the labeling of a word presupposes understanding a deeper agreement between communicators about the way the word is being used. In relation to this he advocated the anti-essentialist notion of family-resemblance; the idea that categories are graded in nature, with better-or-worse exemplars. Against this backdrop of normative agreements, we can do things with language. Performatives such as *I declare you man and wife*, *You are now under arrest*, *I condemn you to prison*, *I name this child*, *I promise to pay the bearer*, impinge on the world because of the mutually held belief between members of a group that in certain contexts these words have force (Austin, 1962; Searle, 1969).

Despite the influence of Wittgenstein, essentialist thinking was still alive and well in much of twentieth century linguistics: the idea that there is an essence of a word that could neatly fit into a lexicon, rather than seeing words as instantiated in a vast network of encyclopedic knowledge (Elman, 2009); the idea that there is an essence of language that all speakers of the same language share, rather than an overlapping set of intuitions defined by the linguistic experience of the individuals (Dąbrowska, 2012); the idea that there is an universal essence to grammar (justifying a distinction between competence and performance), rather than acknowledging the diversity across languages (Bowerman and Choi, 2001; Evans and Levinson, 2009).

Following Popper (1957), evolutionary theorist Mayr (1942) suggested the antidote for essentialism is “population thinking” – a profile of characteristics across a group – just as Wittgenstein had discussed with the idea of family resemblance. It was this kind slipperiness in language that ultimately frustrated the Logical Positivists but which usage-based approaches have turned it into a productive research agenda: prototypes, conceptual metaphors, and probabilistic approaches to syntactic and lexical processing (Ellis, 2002; MacDonald and Christiansen, 2002; Jurafsky, 2003) and grammatical continua, for example, from more verb-like to less noun-like (Harris, 1981; Taylor, 2003).

## Successes, Challenges, and Debates for the Usage-Based Approach

From the evidence reviewed here, the cognitive commitment of usage-based linguistics “to render accounts of syntax, morphology, phonology, and word meaning consonant with aspects of cognition” has accounted for a large range of empirical findings across the domains of language acquisition, processing, and typology. It is of course not without its weaknesses and is there is by no means a consensus as to what usage-based theory is or should be. Here we reflect on what some these debates are, which leads to the final section on where usage-based theory might go from here.

Perhaps unsurprisingly some of the strengths of usage-based theory are also related to its weaknesses; what it gains in breath of explanation it sometimes compromises in depth, for example being able to accommodate a large range of cross-linguistic findings means that stipulating in any detail, *a priori*, which processing constraints and input properties are most important, and when, is very difficult. The problem can be particularly acute in language-acquisition research. The hope is, that once enough of these types of experiments have been conducted, and the results synthesized, usage-based theory will evolve into something that generates tighter “riskier” predictions in the Popperian sense. This would also go a long way to engaging those from different theoretical approaches to language acquisition who are skeptical of the usage-based approach for this reason – “what pattern of results *couldn’t* a usage-based theory explain.” One kind of approach that seems particularly successful in generating this kind of rigor is combining corpus and experimental methods to answer a focused question about learning (e.g., Goldberg et al., 2004; Dąbrowska, 2008; Bannard and Matthews, 2010). In this approach one can use the distributions in the input as the starting point to generate predictions, and then through experimental manipulation, get closer to establishing some kind of cause-and-effect.

Those working in non-usage-based theory can become frustrated when usage-based theorists argue lexical specificity supports the theory, but then are also not worried by evidence of abstraction, since the child’s knowledge is predicted to become abstract at some point in development anyway. Clear developmental predictions about how the process of abstraction should develop, including which systems should become abstract first, are needed. This would also help counter claims that the theory is discontinuous (lexical specificity first, abstraction later). Clearly related to this issue is being able to model language acquisition as a complex dynamic system. For instance, say a child hears the utterance *The dog tickles Mary*. With no other analog in her experience she treats it as an instance of itself, perhaps even storing it as the chunk *ThedogticklesMary*. One might think of this as all constructions starting life toward the more idiomatic end of the constructional spectrum. However, the next time she hears *The dog tickles Peter* it “counts” as both an instance of *The dog tickles Peter* and an instance of *The dog tickles X*. The constriction has moved from being purely idiomatic to having a productive slot, *X*. The difference between this and *kick the bucket* is that the latter is much less productive; compare *hit the bucket, kick the pail*^1^. After many more examples, the child then hears *The dog tickles Mary* for the second time but now it might count as an instance of *The dog tickles Mary*, *The dog tickles* X, *The Y tickles X*, *agent-action-patient*, *subject verb object*, and so on. So, *the same form* is counted as different instances of different things at different times. The situation is analogous to believing a bat is an instance of a bird and then re-categorizing later it as a mammal, as one learns more about the world. The question is, which level of representation do developmental models count and when? Usage-based theories need more psychologically plausible models of what gets treated as a chunk when, and hence “counted” in any distributional analysis (Arnon, 2011). Dynamic systems theory and connectionist models try to address this issue by designing models that grow and internally reorganize themselves (e.g., Thelen and Smith, 1995) but it remains an outstanding challenge for a descriptively and predictively adequate usage-based theory.

A key part of responding to this challenge will be to specify in greater detail the mechanisms of generalization, specifically a mechanistic account of the dimensions over which children and adults make (and do not make) analogies. As usage-based approaches have argued, relational structure, and mapping between representations is a fundamental psychological process that underpins forming these abstract connections. In terms of linguistic form, we know *variation sets* are powerful cross-sentential cues to generalization – two or more sentences that share at least one lexical element, for example, *look at the bunny*, *the bunny is brown*, *silly bunny*, *what a cute bunny*, and so on (Küntay and Slobin, 1996; Waterfall, 2006; Waterfall and Edelman, 2009). Longitudinal studies show that infants are better at structurally appropriate use of nouns and verbs that had occurred in their caregivers’ speech within variation sets, compared to those that did not (Nelson, 1977; Hoff-Ginsberg, 1986, 1990; Waterfall, 2006). Moreover, the cue is relatively available: between 20 and 80% of utterances in a sample of English CDS occur within these variation sets – depending on the threshold of intervening sentences and therefore the definition of “set” (similar statistics apply to Turkish and Mandarin, Küntay and Slobin, 1996; Waterfall, 2006). Clearly generalizations are made not just over form but over meaning also (even if the forms share nothing in common) and here there is less consensus on what counts as a variation set of meaning or even how to formalize one. Usage-based approaches might respond by saying that the meaning is the sum total of how the form is used in a communicative context, however for those seeking more detail, usage-based theorists need to provide a more mechanistic accounts that integrates semantic and formal generalizations. One way to tackle this is to ask, where does the meaning of linguistic form *x* come from in the child’s environment? For example, in many of the world’s languages grammatical aspect is used to indicate how events unfold over time. In English, activities that are ongoing can be distinguished from those that are completed using the morphological marker -*ing*. Using naturalistic observations of children, Ibbotson et al. (2013b) quantified the availability and reliability of the imperfective form in the communicative context of the child performing actions. They found two features of the communicative context reliably mapped onto the functions of the imperfective, namely, that events are construed as ongoing and from within. In theory grammatical aspect is potentially an abstract notion, however, this shows how the pragmatic and referential context in which a child hears language may serve to limit the degrees of freedom on what linguistics constructions mean and therefore the directions in which analogies are made (see also Ibbotson, 2011).

“*The* usage-based” approach is of course something of a generalization in itself; there are almost as many usage-based theories as there are theorists. A major contention in the field, which points to future research, is over what level of representation best characterizes usage patterns. For example, although Ninio (2011) and Goldberg (2006) agree on many of the cognitive principles that underpin usage-based approaches, they radically disagree about the role of semantic similarity in generalization and what are the fundamental units of grammar. Namely, whether they are best characterized as merge-dependency couplets or constructions. Schematic constructions like *X verbs Y* are by no means deemed necessary for all usage-based accounts either. For example, Daelemans and van den Bosch’s (2005) radically exemplar-based computational model, which is still very much rooted in the cognitive linguistic, usage-based approach, questions the necessity of schematic knowledge. The implication is that a large amount of linguistic knowledge may consist of rather specific, low-level knowledge, ultimately instantiated in the memory traces of specific instances. Alternatively, some cognitive grammars posit a degree of schematicity, but only to the extent that they are schematic for actually occurring structures and actually occurring instances. Croft (2001) argues that such things as subjects and DO only exist in constructions (not as schemas that exists as themselves) and are different entities when they appear in different constructions, for example a transitive-subject, a passive-subject, and there-subject. In general though, what unifies usage-based models is a general skepticism about underlying structures that diverge from surface structure with respect to the ordering and content of constituent parts (Langacker, 1987).

Another potential area of development relates to the interface between social-cognitive representations and grammatical representations. There is a lot of work that has emerged in recent years on infants’ development of social cognition and pragmatic reasoning, but it has yet to be worked through in any detail how that understanding interacts with and/or constrains syntactic representations and generalizations. It seems important then that usage-based approaches start integrating the wealth of experimental data on social cognition with the statistical syntactic learning literature. One area that seems particularly relevant here is the grammar-attention interface. For example, Ibbotson et al. (2013a) investigated whether children (3- and 4-year-olds) and adults can use the active/passive alternation – essentially a choice of subject – in a way that is consistent with the eye-gaze of the speaker. Previous work suggests the function of the subject position can be grounded in attentional mechanisms (Tomlin, 1995, 1997). Eye-gaze is one powerful source of directing attention that we know adults and young children are sensitive to; furthermore, we know adults are more likely to look at the subject of their sentence than any other character (Griffin and Bock, 2000; Gleitman et al., 2007). They demonstrated that older children and adults are able to use speaker-gaze to choose a felicitous subject when describing a scene with both agent-focused and patient focused cues.

Taking the “developmental cognitive linguistics” enterprise to its logical conclusion, one of the big questions is whether there are any purely linguistic representations or can all linguistic categories be traced back or decomposed into the functional or communicative roles they play. In the next section we outline why there might be fundamental limits of what we can explain with a purely cognitive conception of usage-based processes.

## Culture and Use

Overall it might be argued that, while significant challenges remain, the usage-based commitment to domain-general cognitive processes has been able to account for a large range of empirical findings across the domains of language acquisition, processing, and typology. Below we briefly sketch out some examples that do not seem to fit easily into this category and present a challenge for a cognitive process level of explanation.

Several authors have argued that we need to acknowledge language as part of a broader species-specific adaptation for cultural life in general, with linguistic norms as specific coordination solutions to the general problem of coordination (Clark, 1996; Tomasello, 1999, 2008; Everett, 2012). It is important to outline what one thinks is special about humans as many of the usage-based claims about core cognitive processes in language – categorization, chunking, rich item-based memory – are likely to be true of other species (and computers). However once our view of language, and more generally of communication, is one of a social act in the context of normative agreements and cooperative reasoning, it is possible to say why domain-general cognitive processes and even species-general processes might be necessary but not sufficient for language.

### Groupishness and normativity

People see themselves as belonging to families, cliques, nations, clans, religions – and of course languages. Being part of a group comes with expectations about how one *ought* to act and think – norms. For example, I tacitly expect other English speakers to put adjectives before the noun they modify. And if they don’t I might tell them that they *should*.

Cross-culturally, people seem particularly adept at reasoning about normative matters compared to non-normative matters (Cosmides and Tooby, 2008). By 3-years-of-age children are better at reasoning about violations in deontic conditionals, e.g., if x then y must/should do z, than they are with indicative conditions, e.g., if x then y does z (Cummins, 1996a,b; Harris and Núñez, 1996). We also know young children from diverse cultural backgrounds “overimitate” adults’ behavior, for instance copying non-functional means to a goal-directed action (Lyons et al., 2007; Nielsen and Tomaselli, 2010). By comparison, Chimpanzees in same situation drop the unnecessary steps and focus on the goal (Tennie et al., 2009; Whiten et al., 2009). This is important as the ability to produce high fidelity copies of cultural tools is a necessary precursor for cumulative cultural evolution to work: if you can’t copy a wheel then you will have to wait for someone to reinvent it. Moreover, the advantage of a general adaptation to imitate is that it doesn’t need to specify “copy what works” because the fact that enough adults are doing it shows at the very least it works well enough for them to still be alive (on average). Chimpanzees seem to exhibit some evolutionary precursors of normative cognition (tolerant societies, well-developed social-cognitive skills, empathetic competence) but appear to lack the shared intentionality that would convert quasi-social norms into human-like collectivized norms (Tomasello and Rakoczy, 2003; Rudolf von Rohr et al., 2011). Thus it seems natural selection has favored some important elements of the architecture of normative cognition – a disposition to learn prevalent norms (imitation) to comply with norms and enforce them (Boyd and Richerson, 1992; Henrich and Boyd, 2001; Boyd et al., 2003; Chudek and Henrich, 2011).

### Cultural constraints on grammar

What is really interesting for the present discussion is when culture and a normative cognition start to interact in a way that starts to constrain grammar. Kulick (1992, p. 2) explains an example of where a linguistic niche is acquired and maintained through normative cognition and is clearly grammatical in nature. Kulick describes New Guinean communities that have “purposely fostered linguistic diversity because they have seen language as a highly salient marker of group identity…[they] have cultivated linguistic differences as a way of “exaggerating” themselves in relation to their neighbors … One community [of Buian language speakers], for instance, switched all its masculine and feminine gender agreements, so that its language’s gender markings were the exact opposite of those of the dialects of the same language spoken in neighboring villages; other communities replaced old words with new ones in order to “be different” from their neighbors’ dialects.”

Kulick (1992) also gives examples from the Selepet speakers of a New Guinean village who, overnight, decided to change their word for “no” from *bia* to *bune* explaining that they wanted to be distinct from other Selepet speakers in a neighboring village. The Buian gender agreement example does not sit easily in any of the core usage-based cognitive processes of categorization, analogy, chunking, and so on, that featured so prominently in the previous sections – at least not in a way that is predictive of the Buian structural change. Instead of principles of communicative efficiency, ease of understanding, informational organization, and so on we have to appeal to a different source of linguistic structure to explain the patterns we see, namely the kind of normative reasoning and cultural learning we discussed above (swopping gender agreement to be different from your neighbors does not really have anything to do with communicative efficiency, indeed, in the short term it probably reduces efficiency). So, the interesting examples are where grammatical structure itself is being shaped by culture and this has important implications for explaining linguistic patterns within usage-based approaches. Below we briefly introduce three examples:

1. When a concept is sufficiently prominent in a culture, that is part of the shared values, it can predict what is left unsaid, as in the case of Amele a language spoken in New Guinea. It is an SOV language but when the meaning of “giving” is expressed the verb is omitted. For example

**Table:** 

*Jo*	*eu*	*ihaciadigen*
House	that	show
‘*I will show that house to you*’		

The verb *ihac* means “to show” but in the example below the verb is omitted

**Table:** 

Naus	Dege	houten
Name	name	pig
‘*Naus [gave] a pig to Dege*’		
(Everett, 2012, p. 194)		

Roberts (1998) argues that there is no verb “to give” because giving is so basic to Amele culture that this can be left backgrounded.
2. Speakers of Wari’, an Amazonian Indian language, report on others’ thoughts, character, reactions, and other intentional states by means of a quotative structure. Regardless of whether people say something or not they are quoted as saying so in order to communicate what the speaker believes they were thinking. Everett (2012) argues this structure can be traced back to, and explained by, the way Amazonian Indians use a metaphor of “motives” and “will,” as in “*the sky says it is going to rain*,” “*the Tapir says it will run from me*,” and “*John said he was tired of talking with us*,” even when John did not say this, but he behaved as though he did. Because of this the verb “to say” is omitted and the quotation structure (capitalized below) in Wari’ acts as the verb:

**Table:** 

MA’ CO MAO NAIN GUJARÁ nanam ‘oro narima, taramaxicon
‘Who went to the city of Guajará?’ [said] the chief to the women.’
(Everett, 2012, p. 196)

In Wari a high cultural value is placed on evidence for beliefs and since intentions require first-hand evidence this created a linguistic niche filled by the quotative structure which does not commit the speaker to first-hand knowledge.
3. Relatedly, Pirahã, another Amazonian language, requires evidence for assertions – a declarative has to be witnessed, heard from a third party or reasoned from the facts. Pirahã marks this with a suffix, for example – hiai (hearsay), sibiga (deduced or inferred), and xáágahá (observed). The verb and the objects implicated by the verb are obliged to be licensed by the verb’s evidential suffix. The culture places high value on evidence for declarations, which in turn is realized on the evidential suffix, which in turn controls the verbal frame. Because in most languages evidentials are limited to main clauses, the claim is that embedding clauses (recursion) is not possible in Pirahã on a clausal level – as a subordinate clause would not be licensed by the evidential, violating the cultural/grammatical constraints.

There are other intriguing lines of research that show how culture impinges on grammar, for example, *Deixis, grammar, and culture* (Perkins, 1992) describes an inverse correlation between the technological state of a culture and the complexity of their pronoun system. More generally, the key point here is that there exists a pattern of structural use, omission, and agreement that is difficult to predict without expanding the circle of explanations to include cultural as well as cognitive factors. The interpretations of these patterns are controversial (Corballis, 2011) and riddled with potential confounds but they do point to level of linguistic analysis that might be beyond usage-based cognitive principles.

The same argument can be applied at different levels of granularity depending on which source of cultural variation we are focused on. We have known since the work of Labov and Trudgill that heterogeneity in language structure correlates with socio-linguistic variables of class, social network, and ethnicity, for example – all of which could analyzed within a normative framework. What this means is that usage-based cognitive constraints *permit* a possible space of languages but might not *entail* any particular language – the boundaries of the space of cognition do not make contact with that of language in way that allows those predictions. The rather weak predictive synthesis between these domains is in some sense a consequence of the “cognitive commitment” which seeks to make linguistics “merely” *consonant* with aspects of cognition. The integration of cultural constrains with cognitive constraints will place further limits on the space of possible and impossible languages, creating tighter predictions. This would be an example of reductionism – or consilience to use E. O. Wilson’s term – in the better sense of the word: where each domain of understanding is grounded in the next but with no domain being dispensable and where the emphasis is on unifying, integrating or synthesizing aspects of knowledge.

## Conclusion

Usage-based theories of representation see language as a complex adaptive system; the interaction between cognition and use. Findings from language acquisition research, typology, and psycholinguistics are converging on the idea that language representations are fundamentally built out of use and generalizations over those usage events. Interestingly, none of the fundamental mechanisms of the usage-based approach are required to be a language-specific adaptation. Language shows creativity, categories, and recursion because people think creatively, categorically, and recursively. Here we have also drawn special attention to a set of culturally generated structural patterns that seem to lie beyond the explanation of the core usage-based cognitive processes. As well as addressing the need for greater clarity on the mechanisms of generalizations and the fundamental units of grammar, we suggest one of the next big challenges for usage-based theory will be unify the anthropological linguistic data with usage-based cognition.

## Conflict of Interest Statement

The authors declare that the research was conducted in the absence of any commercial or financial relationships that could be construed as a potential conflict of interest.

## Footnotes

^1^In fact even this idiom shows some flexibility in tense: *he is going to kickØ the bucket* vs. *he has kicked the bucket*.
